# Dry season soil water potential maps of a 50 hectare tropical forest plot on Barro Colorado Island, Panama

**DOI:** 10.1038/s41597-019-0072-z

**Published:** 2019-05-17

**Authors:** Stefan J. Kupers, Christian Wirth, Bettina M. J. Engelbrecht, Nadja Rüger

**Affiliations:** 1 German Centre for Integrative Biodiversity Research (iDiv) Halle-Jena-Leipzig, Deutscher Platz 5e, 04103 Leipzig, Germany; 2 Systematic Botany and Functional Biodiversity, Institute of Biology, University of Leipzig, Johannisallee 21-23, 04103 Leipzig, Germany; 3 Max-Planck-Institute for Biogeochemistry, Hans-Knöll Str. 10, 07745 Jena, Germany; 4 Department of Plant Ecology, Bayreuth Center of Ecology and Environmental Research (BayCEER), University of Bayreuth, Universitätsstrasse 31, 95447 Bayreuth, Germany; 5 Smithsonian Tropical Research Institute, Apartado, 0843-03092 Balboa, Ancón Panama

**Keywords:** Hydrology, Forest ecology, Tropical ecology

## Abstract

Fine scale spatial variation in soil moisture influences plant performance, species distributions and diversity. However, detailed information on local soil moisture variation is scarce, particularly in species-rich tropical forests. We measured soil water potential and soil water content in the 50-ha Forest Dynamics Plot on Barro Colorado Island (BCI), Panama, one of the best-studied tropical forests in the world. We present maps of soil water potential for several dry season stages during a regular year and during an El Niño drought. Additionally, we provide code that allows users to create maps for specific dates. The maps can be combined with other freely available datasets such as long-term vegetation censuses (ranging from seeds to adult trees), data on other resources (e.g. light and nutrients) and remote sensing data (e.g. LiDAR and imaging spectroscopy). Users can study questions in various disciplines such as population and community ecology, plant physiology and hydrology under current and future climate conditions.

## Abstract (summary)

**Table:** 

Design Type(s)	modeling and simulation objective • data collection and processing objective
Measurement Type(s)	wetness of soil
Technology Type(s)	computational modeling technique
Factor Type(s)	season
Sample Characteristic(s)	Barro Colorado Island • primary forest

Machine-accessible metadata file describing the reported data (ISA-Tab format)

## Background & Summary

Water is an essential resource for plants and is crucial for numerous plant functions^1^. Consequently, water availability strongly influences plant performance, species distributions, functional composition and ecosystem functioning across biomes^2–6^. On local scales, spatial variation in soil moisture differentially affects performance among species^7–9^, promoting niche differentiation in plant communities and fostering coexistence^10^. Understanding how local soil moisture variation affects plants will become increasingly important, given the predicted shifts in rainfall patterns caused by climate change and their expected effects on plant performance, community composition and species distributions^2,4,11^.

In tropical forests, local variation in soil moisture causes tree species to perform differently among habitats^12–14^, which promotes habitat associations and may contribute to the maintenance of high species diversity in these forests^8,15,16^. However, soil moisture also affects species performance and distributions at smaller scales than habitats, highlighting the importance of measuring fine-scale spatial variation in soil moisture^9^. Most studies that link species performance to soil moisture have measured soil water content^17–20^. Yet, soils with similar water contents can differ in characteristics that influence the availability of water for plants, such as texture, bulk density and pore size distribution^21,22^. A more relevant measure for plant-water relations is soil water potential, because plants extract water from the soil along a soil-plant-air gradient of water potential^23^.

**Fig. 1:**
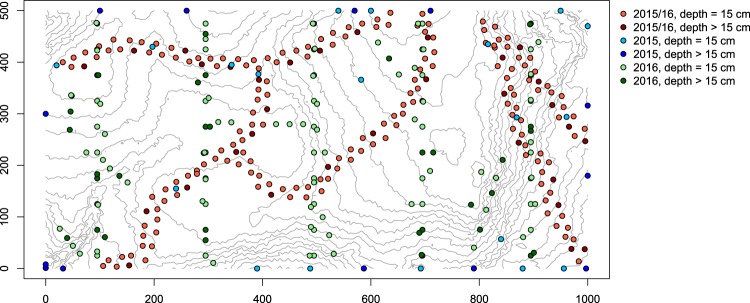
Sampling locations and depth of soil sampling in the 50-ha plot on BCI, Panama, during the dry seasons of 2015 and 2016. Samples >15 cm depth within the 50-ha plot and around the plot perimeter were taken down to a depth of 40 cm and 100 cm, respectively. Intervals of the contour lines are 2 m.

We measured dry-season soil water potential and soil water content across a 50-ha Forest Dynamics Plot on Barro Colorado Island, Panama (Fig. 1). The plot consists of seasonal lowland tropical forest, a forest type that occurs in large parts of the tropical regions of Africa, Asia and Latin America^24^. The 50-ha plot was established in 1981 and is the first plot in the global CTFS-ForestGEO network^25,26^. Regular censuses document the entire life cycle from seeds to adults for more than 300 species of trees and climbers, making it one of the best-studied tropical forests in the world^24,26–28^. Because of the strong seasonality and inter-annual variation in rainfall and soil moisture on BCI, we measured soil moisture across several stages of a normal dry season and a dry season associated with a severe El Niño drought^29^. We used Random Forests^30^ to model spatial variation in soil water potential across the 50-ha plot on a 5 m resolution during various stages of the dry season, using soil monitoring data to quantify drought intensity, as well as topographic and edaphic information and data from a tree census (see Table 1). We provide the original soil moisture data and adjustable code that allows users to create custom maps for any date in the dry season since 1975 and to apply different model settings or algorithms.

**Table 1:** Variables used in the Random Forest models.

Variable	Type	Source
Elevation	Spatial (horizontal)	ForestGEO^46^
Slope	Spatial (horizontal)	ForestGEO^46^
Soil type	Spatial (horizontal)	Baillie *et al*.^44^ and Harms *et al*.^43^
ln(Basal area) of trees ≥1 cm dbh in the 2015 census	Spatial (horizontal)	Hubbell *et al*.^36^
ln(Depth) of soil sampling	Spatial (vertical)	Soil sampling records^52^
Time of soil sampling	Temporal	Soil sampling records^52^
Monitored soil water content	Temporal	Smithsonian Tropical Research Institute^47^

The approach we developed generates soil water potential maps at very high spatial and temporal resolution (i.e. 5 m resolution for any day). These data, therefore, are ideal for studies that focus on ecological or hydrological processes on a local scale^1,31^. In addition, they complement soil moisture estimates from satellite data, which are ideal for upscaling local measurements to regional scales (e.g., across a climatic gradient). Recently launched satellites such as Sentinel 1 and 2 have the potential of estimating soil water content on a maximum resolution of 100 m^32,33^. In the future, these high-resolution soil moisture products from Sentinels can be compared with our *in-situ* measurements.

The maps can be combined with various datasets collected in the 50-ha plot, such as surveys of light availability^34^ and soil chemistry^35^, long-term censuses of flowers, seeds and seedlings^28^ and trees^36^, and detailed remote sensing datasets such as airborne imaging spectroscopy^37^ and light detection and ranging (LiDAR) data^38^. Users may explore the role of soil moisture in various fields of research such as community assembly, niche differentiation and coexistence, hydrology and nutrient transport, and soil carbon cycling and storage. In addition, the maps of various dry season conditions can be used to plan new observational studies, to quantify the effect of climate variability (such as El Niño droughts) on the performance and distribution of tree species and to predict the effect of expected shifts in rainfall patterns caused by climate change^11^.

## Methods

### Study site

The 50-ha Forest Dynamics Plot on Barro Colorado Island, Panama (9.15°N, 79.85°W), supports semideciduous lowland moist tropical forest^39^. Most of the plot is old growth forest (>300 years old), except for 2 ha that is in late secondary succession (>100 years old)^26^. Rainfall is strongly seasonal: only 10% of the 2660 mm annual precipitation falls in the dry season from mid-December to late April^29^. The intensity and length of the dry season are highly variable, and dry seasons during El Niño events tend to be particularly long^40^.

The topography of the 50-ha plot is relatively flat with slopes ranging from 0 to 21 degrees, and elevation ranging from 120 to 155 meters asl^26^. Soil water availability is higher (i.e. soil water potential is less negative) on slopes than on plateaus^41,42^ due to the geology and hydrology of the plot; the water table is close to the surface and creates several springs on the slopes, and water drains via the slopes that form the edges of an andesite cap with low permeability underlying the high plateau^39,41,43^. There are four types of red clay soils defined in the local soil classification system for BCI: AVA covers most of the flat terrain across the plot, Marron covers the eastern slopes and parts of the low plateau, Fairchild covers the southeast corner of the plot and Swamp covers the central depression^44^. The soils drain freely except the Swamp soil and parts of the AVA soil, which encounter seasonal flooding in the wet season^26,44^. Soil water availability at 20 cm depth tends to be higher in gaps than in the understory, although shallower soils may be drier in gaps^41^. Wetter subsurface soils in gaps are likely caused by concentrated rainfall as drip lines from the edges of tree crowns, as well as higher rainfall in gaps and lower root density which decreases water extraction from the soil^41^. More detailed descriptions of the plot are given in Condit^39^.

### Soil moisture sampling

**Fig. 2:**
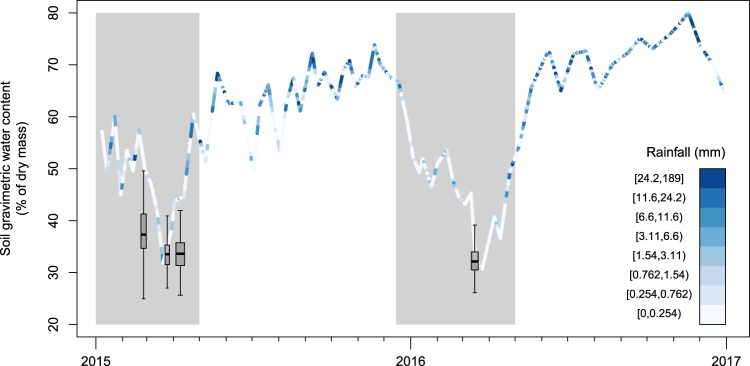
Soil water content (SWC) measured in the 50-ha plot in the four sampling periods (box and whisker plots) and monitored 1.25 km from the 50-ha plot (lines). The width of the boxes represents the number of days in the sampling period (see Methods). Colours indicate daily rainfall. Grey shading indicates dry seasons. Rainfall and SWC monitoring data are from the Smithsonian Tropical Research Institute (STRI) Physical Monitoring Program^47^.

We collected soils during three periods in the 2015 dry season (February, March, and April) and one period in the 2016 dry season (March) (Fig. 2). The sampling periods were 6, 5, 10 and 8 days long, respectively. The 2016 dry season was associated with the 2015–2016 El Niño, and was the third longest dry season on BCI since 1954^29^. We took samples at a total of 363 sites, consisting of 200 seed trap sites along the trails of the 50-ha plot^45^ and 163 other sites in the plot and around its border (Fig. 1). To reduce disturbance of the vegetation in the plot, we took most samples at the easily accessible seed traps: in all four sampling periods, we took one sample at 15 cm depth at each seed trap^45^. The seed traps cover all soil types^44^ and major habitats in the plot except streamsides (cf. Harms *et al*.^43^). In April 2015 and in 2016 we took samples down to 40 cm depth at 100 sites along north-south transects in the plot, as well as at 41 sites with steep slopes (>15°) or rare habitats such as treefall gaps, the swamp and streamsides. Additionally, we took samples down to 100 cm depth at 22 sites around the plot perimeter in the three sampling periods in 2015. In total, we took 1299 samples that covered all soil types and habitats in the plot (Table 2). Finally, we assessed small-scale variation in soil moisture at eight seed traps by taking samples at 15 cm depth at the trap and two samples per distance class from the trap (1, 2 and 4 meters) in random compass directions.

**Table 2:** Number and percentage of samples taken in each habitat and soil type, and the percentage of the plot area covered by each habitat and soil type.

		# of samples	% of samples	% of plot area
Habitat	High plateau	93	7.2%	13.6%
	Low plateau	669	51.5%	49.6%
	Mixed	55	4.2%	5.3%
	Slope	292	22.5%	22.7%
	Stream	46	3.5%	2.6%
	Swamp	25	1.9%	2.4%
	Young	119	9.2%	3.8%
Soil type	AVA	796	61.2%	71.9%
	Fairchild	46	3.5%	0.8%
	Marron	429	33.0%	25.0%
	Swamp	28	2.2%	2.3%

We collected the soil samples with 1–3 cm diameter soil augers, depending on the sampling depth. We inserted the auger into the soil until the depths mentioned above. We sealed the lowest 1 cm of the soil core in airtight plastic vials for soil water potential measurements and the 9 cm above it in zip lock bags for soil water content measurements. Then we transported the samples to the laboratory in insulating containers with cooling elements. In the lab, we measured soil water potential (SWP) for each sample with a WP4C Dewpoint PotentiaMeter (Decagon Devices, Inc., Pullman WA, USA). We also assessed soil water content (SWC) gravimetrically for each sample from fresh mass (f) and dry mass (d) determined after 72 hours at 105 °C (SWC = (f − d)/d).

### Predictors

We used seven predictors to model SWP throughout the 50-ha plot (Table 1). We derived elevation and slope on a 5 m resolution from a digital elevation model^46^. We also digitized a map of soil type on a 5 m resolution from a survey report on BCI soils^44^. On the coarse soil survey map, the seasonal swamp was shifted northwards compared to the more detailed habitat map of Harms *et al*.^43^. We assigned the Swamp soil type to the area defined as swamp in the habitat map and assigned the soil type surrounding the swamp (Marron) to the area north of the newly defined swamp. Additionally, we summed and ln-transformed basal area in each 5 × 5 m subquadrat for all trees ≥1 cm diameter at breast height in the 2015 tree census^36^ to account for the effect of vegetation density and treefall gaps on water availability. We also accounted for variation in SWP caused by the ln(depth) and time of sampling.

To assess temporal variation of SWP caused by differences in drought intensity, we used SWC monitoring data collected by the Smithsonian Tropical Research Institute every one to two weeks at 10 locations in a catchment 1.25 km from the 50-ha plot^47^. We calculated the mean SWC for each monitoring day, and calculated SWC for our sampling days by linear interpolation between SWC of the monitoring days. Although SWC from our soil samples was slightly lower than monitored SWC, probably due to different soil types and flatter terrain in the 50-ha plot, the temporal trend was similar (Fig. 2). Monitored SWC also accounted for rainfall during sampling. There were four days with light showers on BCI during the sampling periods, two of which had sufficient rain (5 mm on 8 April 2015 and 1 mm on 17 March 2016) to reach the forest floor (>0.5 mm)^48^. Monitored SWC increased in the week of 8 April 2015 and started to decline less steeply in the week of 17 March 2016 (Fig. 2). We compared SWP predictions using monitored SWC versus cumulative water deficit (a water balance based on rainfall and evapotranspiration) as an alternative indicator of drought intensity. We found that monitored SWC captured the severe drought in 2016 well whereas cumulative water deficits in 2016 were less negative than expected, probably due to the incomplete saturation of the soil during the previous wet season.

### Random Forest modelling and mapping

We modelled SWP using Random Forests (RF)^30^. RF is a machine learning method that aggregates many decision trees (simple models that use binary splits to relate a response to predictors) that are constructed with a bootstrapped sample of the data for each tree and a random subset of the predictors^49^. RF performs well relative to similar algorithms and is robust to overfitting, noise and uneven spatial sampling^30,49,50^. We compared RF with Boosted Regression Trees, another algorithm known for its high predictive performance^51^. We found little difference in performance but smoother fits between SWP and the predictors in RF compared to BRT, indicating less overfitting in RF.

After assessing goodness of fit (see Technical Validation), we used the RF model to map SWP. For slope, elevation and soil type, we determined the nearest data point to the centre of each 5 × 5 m quadrat in the 50-ha plot. Basal area was set to the median across the plot (0.03 m^2^ per 5 × 5 m quadrat). As soil moisture varies strongly during the dry season, we created maps of soil water potential for various levels of dry season intensity (see Data Records).

## Data Records

**Fig. 3:**
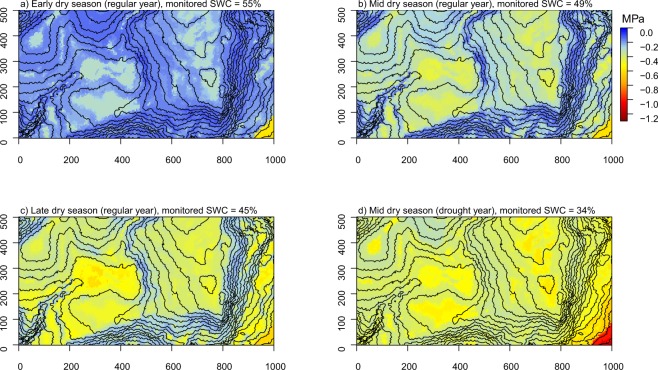
Soil water potential (SWP) in the 50-ha plot on BCI. SWP is modelled with a Random Forest algorithm on a 5 m resolution at 15 cm depth and at 12 PM. SWP is shown for (**a**) early, (**b**) mid and (**c**) late dry season conditions for a regular year, and (**d**) mid dry season during a drought. Monitored soil water contents (SWC) correspond to the (**a**) 25^th^, (**b**) 50^th^ and (**c**) 75^th^ percentiles of monitored SWC during February, March and April from 1972 to 2018, and (**d**) median monitored SWC during our soil moisture measurements in March 2016, which was part of an El Niño drought. Basal area was fixed to the median basal area of the 5 × 5 m quadrats (0.03 m^2^). Intervals of the contour lines are 2 m.

**Fig. 4:**
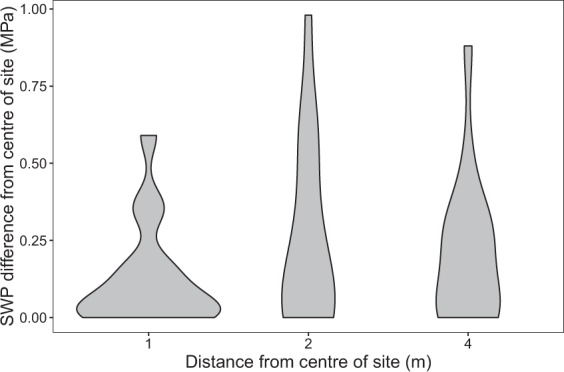
Small scale variation in soil water potential (SWP). Graphs show the difference between SWP in the centre of a site (*n* = 8) and SWP measured at 1, 2 or 4 meters from the centre in a random compass direction. The 10^th^, 50^th^ and 90^th^ percentiles of the differences in SWP per distance are 0.00, 0.06 and 0.36 MPa for 1 m distance, 0.00, 0.14 and 0.61 MPa for 2 m distance and 0.00, 0.15 and 0.41 MPa for 4 m distance, respectively.

All data are freely available from Figshare^52^. We provide soil moisture sampling data and soil water potential maps for early, mid and late dry season conditions during a regular year and for mid dry season conditions during a severe drought (Fig. 3). The maps are provided as pdf files, text files and TIFF images to facilitate viewing, analyses and visualization in various software packages (Table 3). We also provide the Random Forest model and the soil type map we digitized from Baillie *et al*.^44^ for users creating custom maps (Table 3). All other data needed for creating custom maps are freely available through the links in the code. Finally, we provide data on small-scale soil moisture variation (Fig. 4).

**Table 3:** List of data records. All data are available from Figshare^52^.

Filename	Type(s)	Description
BCI_Soil_moisture_mapping	.txt	Soil moisture data used for mapping
BCI_Soil_moisture_small_scale	.txt	Soil moisture data used for assessing small scale variation
BCI_ Soil_moisture_R_code	.R	R code for creating maps
BCI_soil_type	.txt	Soil type map digitized from Baillie *et al*.^44^
BCI_SWP_RF_model	.RData	Random Forest model (rfsrc object in R)
BCI_SWP_map_early_dry_season_regular BCI_SWP_map_mid_dry_season_regular BCI_SWP_map_late_dry_season_regular BCI_SWP_map_ mid_dry_season_drought	.txt,.pdf,.tif	Maps of soil water potential as presented in Fig. 3

## Technical Validation

**Fig. 5:**
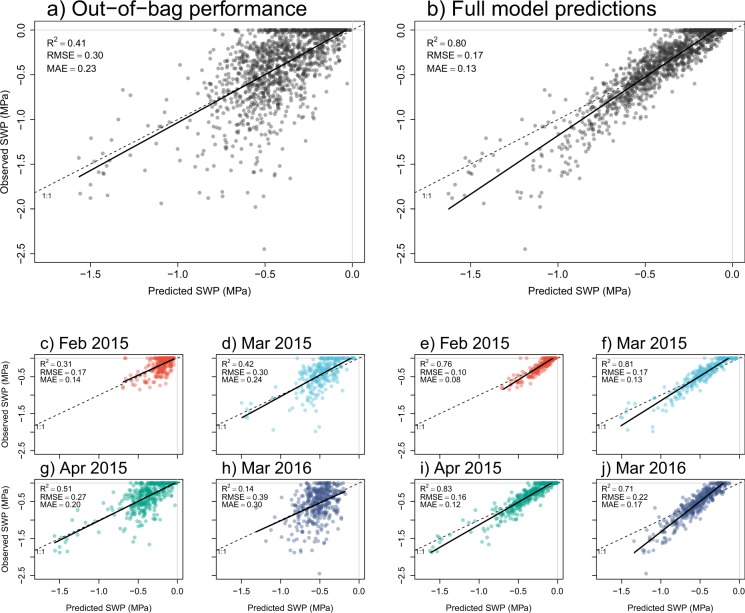
Observed soil water potential (SWP) versus SWP predicted by the Random Forest (RF) model. (**a**) We assessed model performance from the single decision trees that were fitted on a bootstrapped sample to predict SWP for the out-of-bag data (see Technical Validation for details). (**b**) We made predictions of the full model based on the aggregated decision trees. We also show performance (**c**,**d**,**g**,**h**) and predictions (**e**,**f**,**i**,**j**) of the same RF model for the separate sampling periods.

We estimated the goodness of fit of the Random Forest model using out-of-bag (OOB) data, which performs similarly to setting aside a test set^30^. For each bootstrapped iteration, the model used the tree that was created based on the bootstrapped sample to predict SWP for the data that was not in the bootstrapped sample (i.e. the OOB data, comprising around one-third of the observations per iteration)^49^. For each SWP observation, the mean predicted SWP across iterations was used to calculate error metrics^30^. The proportion of variance explained by the model (R^2^) for all sampling periods combined was 0.41, the Root Mean Squared Error (RMSE) was 0.30 MPa and the Mean Absolute Error (MAE) was 0.23 MPa (Fig. 5a). Predictions were particularly accurate for April 2015 (R^2^ = 0.51, Fig. 5g). Predictions were less accurate for February 2015 (R^2^ = 0.31, Fig. 5c) and for March 2016 (R^2^ = 0.14, Fig. 5h). After assessing goodness of fit, we predicted SWP with the full model, i.e. with the aggregated trees (Fig. 5, left panels). Variance explained was higher and errors were lower in the full model compared to the OOB values (Fig. 5b). However, note that the OOB values should be used to estimate model performance^30,49^. The full model slightly overestimated SWP in the lower SWP range (predicted SWP was slightly higher than observed), particularly for the El Niño drought in March 2016 (Fig. 5j). The lowest SWP we measured was −2.45 MPa in March 2016 (see Fig. 5h), which was similar to the lowest value measured in the plot (−2.3 MPa) during the relatively dry 1985 dry season^41^.

**Fig. 6:**
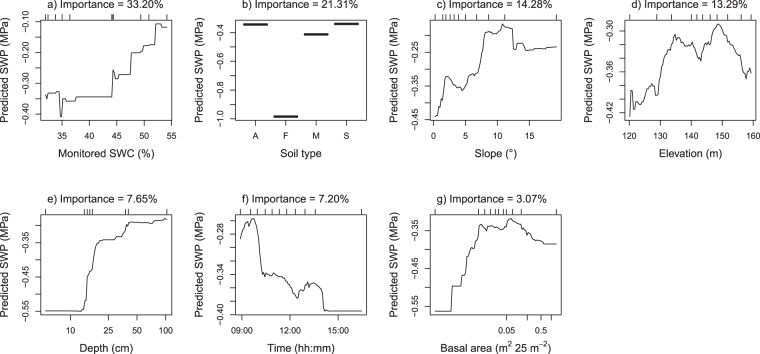
Fitted values of soil water potential (SWP) versus predictors of the Random Forest model. Predictors are sorted based on their importance value. Ticks above the graphs indicate deciles of the predictor observations. Soil types are AVA (A), Fairchild (F), Marron (M) and Swamp (S)^44^.

The importance of predictors and their relationship with SWP was generally as expected (Fig. 6). SWP was most strongly related to monitored soil water content; SWP in the 50-ha plot increased (i.e. soils were wetter) with increasing soil water content at the monitoring location (Fig. 6a). Soil type had a strong influence on SWP as well. Fairchild soil in the southeast corner of the plot was much drier than the other soil types (Fig. 6b). Fairchild soil that we sampled had a distinct white to yellow colour, it drains freely and is the only soil in the 50-ha plot that is not derived from andesite parent material^44^. AVA and Swamp soils were wetter than Marron soil, likely because they encounter seasonal flooding^44^. We expected the swamp to be even wetter than our model predicted (see flat area in the centre of the plot in Fig. 3). There are two likely reasons for the drier predictions in the swamp. First, we took most measurements in the swamp during the severe 2016 dry season (see Fig. 1), when the swamp largely dried out. Second, the swamp is mostly flat, and flat terrain was generally drier than slopes. Higher SWP on slopes (Fig. 6c) corresponded to earlier findings in the plot^41^, indicating that the water table reaches the surface on the slopes around the edges of the relatively impermeable andesite cap under the high plateau^39,41,43^. High and low elevation sites were generally dry (Fig. 6d), likely because these elevations consist of two plateaus that are further from the water table than the slopes that connect them and because of the exceptionally dry Fairchild soil at low elevations.

Depth, time of sampling and basal area had a much weaker effect on SWP. SWP increased in deeper soil layers (Fig. 6d) and decreased in the course of the day as the soil dried out (Fig. 6e). SWP was higher in quadrats with high basal area (Fig. 6g), which contrasts with higher SWP in gaps versus understory measured at 20 cm depth at two locations in the 50-ha plot^41^. However, we measured SWP mostly at 15 cm depth and these surface soils may be drier in gaps^41^. Additionally, soil drying on BCI varies with gap size; evaporation is more important than water extraction from roots in large gaps whereas this is reversed in small gaps and in the understory^53^, indicating that the relationship between canopy structure and soil moisture is complex.

## Usage Notes

In addition to using the presented maps, users can adapt the provided code to produce maps for most dates (approximately from February until April) in any dry season starting from 1975, the year in which consistent monitoring of soil water content was started. The measurements in March 2016 covered the lowest levels of soil water content since monitoring started^47^, so droughts can be mapped as well. The measurements did not cover wet seasons nor very early dry seasons (mid-December or January), so these periods cannot be mapped accurately. Soil water potential during these periods will be mostly saturated (0.00 MPa).

## ISA-Tab metadata file

Download metadata file

## Data Availability

The code was written and annotated in R 3.4.1^54^ and is available from Figshare^52^. The key package for implementing Random Forests was randomforestSRC 2.7.0^55^.
